# Stimuli-Responsive Hydrogels: From Swelling–Deswelling Mechanisms to Biomedical Applications

**DOI:** 10.3390/nano16050329

**Published:** 2026-03-05

**Authors:** Meyoung-Kon Kim, Junghan Lee, A-Ram Kang

**Affiliations:** 1 Department of Biochemistry and Molecular Biology, Korea University College of Medicine, Seoul 02841, Republic of Korea; jerrykim@korea.ac.kr; 2 Department of Biomedical Science, Inha University College of Medicine, Incheon 22332, Republic of Korea; jhlee02@inha.ac.kr

**Keywords:** stimuli-responsive hydrogel, drug delivery, biosensor, tissue engineering

## Abstract

Stimuli-responsive hydrogels, also referred to as “smart” hydrogels, have emerged as versatile platforms for a wide range of biological and biomedical applications owing to their tunable physical, chemical, and biocompatible properties. Their adaptability arises from both their ability to undergo reversible swelling–deswelling and volume phase transitions in response to specific physicochemical or biological stimuli and the diversity of synthesis strategies that enable precise tailoring of material properties to meet distinct biomedical demands. Recent advances have led to the development of novel hydrogel designs with improved swelling–deswelling behavior, enhanced stimulus sensitivity, and superior biocompatibility, thereby expanding their applicability in complex biological environments. Despite this progress, challenges such as precise control over hydrogel size and relatively slow response kinetics remain critical barriers to broader biomedical and clinical translation. Addressing these limitations requires strategies, including reducing hydrogel particle dimensions to accelerate response rates and engineering heterogeneous or highly porous gel architectures to increase functional surface area. This review provides a comprehensive classification of stimuli-responsive hydrogels based on their physical properties and response mechanisms, and summarizes recent innovations in their design, synthesis, and biomedical applications. Furthermore, it discusses emerging approaches to enhance the clinical applicability of smart hydrogels in controlled drug release, targeted gene delivery, biosensor development, and tissue engineering. Overall, continued optimization of swelling–deswelling characteristics and material design will be essential to fully realize the potential of stimuli-responsive hydrogels in precision medicine and advanced therapeutic applications.

## 1. Introduction

Hydrogels are three-dimensional (3D) hydrophilic polymeric structures whose liquid component is water. Hydrogels are produced by a 3D network of natural or synthetic polymers via physical (e.g., ionic, hydrophobic or hydrogen bonding interactions) and/or chemical crosslinking (e.g., polymerization, radiation, small-molecular crosslinking, and polymer–polymer crosslinking). These crosslinking methods enable hydrogels to absorb and retain vast amounts of water—up to thousands of times their dry weight—without dissolving in aqueous solutions. Hydrogels’ elastic, absorbent and permeable nature allows them to hold a substantial amount of water content, closely resembling natural tissue. Consequently, hydrogels are highly sought after for biomedical applications owing to their biodegradability and biocompatibility, which have attracted significant interest in recent decades. Biodegradable hydrogels used in biomedical applications offer a unique advantage: they degrade through the body’s enzymatic and hydrolytic processes once their purpose is fulfilled, eliminating the need for physical removal. Synthetic hydrogel polymers, however, face challenges due to limited biocompatibility and biodegradability, which initially restricted their use in tissue regeneration. To overcome these limitations, substantial efforts have been made to modify hydrogel properties, enhancing their functionality for various biomedical and tissue engineering applications.

In particular, stimuli-responsive polymeric hydrogels, i.e., ‘smart’ hydrogels, have great potential for biological and medical applications due to their enhanced physical, chemical and biocompatible properties [1,2,3,4]. These smart hydrogels can respond to specific environmental stimuli through gel-sol transitions or volume phase changes. Stimuli can be either physical (light, temperature, electricity, magnetic or pressure) or chemical (pH, ionic strength, or other biochemical materials). Numerous stimuli-sensitive hydrogels have been developed and tested for biomedical applications [5,6,7,8,9,10,11,12,13]. Stimuli-responsive hydrogels can transform in response to specific environmental cues. Physical stimuli include light, temperature, electricity, magnetic fields, and pressure. For example, light-responsive hydrogels contain photo-responsive substances that induce volume changes when exposed to specific wavelengths of light (e.g., ultraviolet), leading to chemical or physical transformations in the hydrogel network. Thermo-responsive hydrogels expand or contract within a certain temperature range, undergoing phase transitions at either a Lower Critical Solution Temperature (LCST) or an Upper Critical Solution Temperature (UCST). Electro-sensitive hydrogels undergo volume or shape changes when exposed to an electric field, while magneto-responsive hydrogels contain magnetic nanoparticles that induce mechanical or volume changes under a magnetic field. Pressure-responsive hydrogels alter their structure and physical properties when mechanical pressure or stress is applied.

Chemical stimuli for hydrogels include pH, ionic strength, and biochemical substances. pH-responsive hydrogels expand or contract within a specific pH range; their acidic or basic groups within the polymer network respond to pH changes by altering their ionization state, leading to volume adjustments. Ionic strength-responsive hydrogels react to changes in ion concentration, with charge interactions between polymer chains influencing volume in response to varying ion levels. Biochemical substance-responsive hydrogels interact with specific biomolecules such as enzymes, glucose, or proteins through bio-recognition elements that induce changes in the hydrogel in the presence of these substances.

In this review, we aim to bridge the gap between molecular swelling–deswelling mechanisms and practical biomedical implementation. Rather than providing a purely descriptive classification, we organize stimuli-responsive hydrogels according to their dominant stimulus mechanisms—temperature, pH, electric, and biomolecular—and critically evaluate how their physicochemical response principles influence design strategies, functional performance, and translational feasibility. By framing hydrogel behavior within a mechanistic and application-oriented context, we provide a structured perspective to guide future material innovation and clinical development.

## 2. Types of Stimuli-Responsive Hydrogels

### 2.1. Temperature-Sensitive Hydrogels

Temperature-sensitive hydrogels are among the most extensively studied smart hydrogels due to their ability to undergo reversible sol–gel transitions near physiological temperatures. This property has positioned thermoresponsive hydrogels as particularly attractive candidates for injectable and in situ-forming systems, enabling minimally invasive administration and localized therapeutic action.

**Figure 1:**
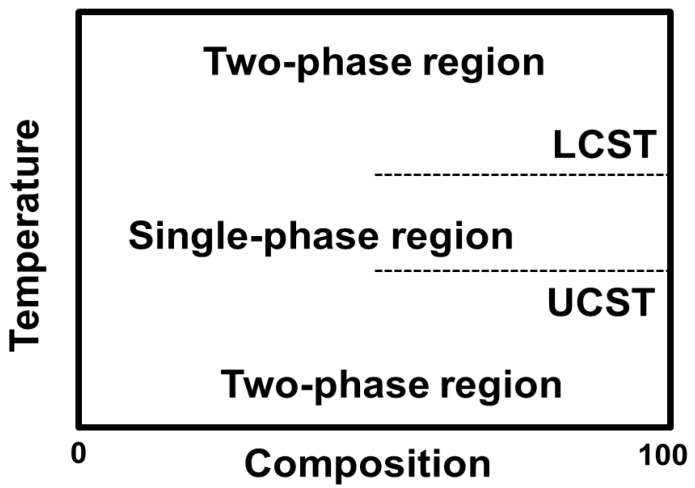
Phase diagrams of LCST- and UCST-type polymers.

**Figure 2:**
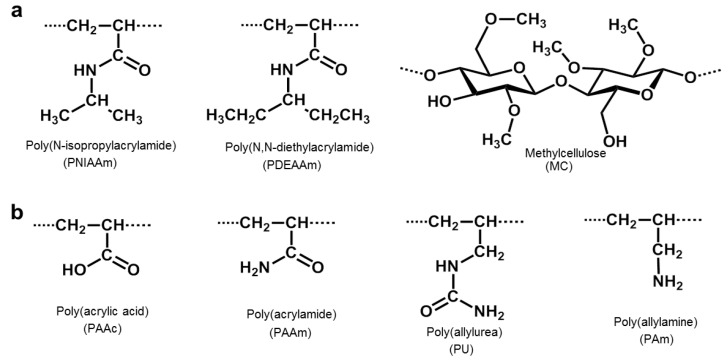
Polymers with LCST (**a**) and UCST (**b**).

The sol-to-gel phase transition temperature can be lower, known as LCST, or higher, known as UCST [14,15]. LCST hydrogels mix well with the solvent below a specific temperature, where hydrogen bonding between hydrophilic segments increases, promoting polymer dissolution. This bonding explains why hydrogels expand below the LCST. However, at temperatures above the LCST, hydrogen bonding between hydrophilic segments decreases, making the polymer insoluble and causing it to precipitate. At the molecular level, swelling–deswelling transitions are governed by the dynamic balance between polymer–water hydrogen bonding and hydrophobic interactions within the network. Below the LCST, hydrogen bonding stabilizes the hydrated structure, whereas above the LCST, entropy-driven dehydration and enhanced hydrophobic association lead to polymer chain collapse and macroscopic volume shrinkage [16]. In ionizable systems, electrostatic interactions further modulate osmotic pressure, influencing network expansion or contraction. While these molecular mechanisms define the fundamental phase behavior of thermoresponsive hydrogels, their clinical and translational relevance depends on how efficiently such transitions occur within complex biological environments [17]. In bulk systems, diffusion-limited water transport and structural heterogeneity may delay or dampen deswelling responses, thereby limiting spatial precision in drug delivery applications [17,18]. Consequently, recent design strategies increasingly focus on nanoscale architectures, hierarchical porosity, and compositional tuning to optimize response kinetics without compromising mechanical stability. In contrast, UCST hydrogels swell at temperatures above the UCST. Figure 1 shows the phase diagrams of polymers with LCST and UCST as previously described [14]. Hydrophobic functional groups, such as methyl, ethyl and propyl groups, confer temperature-specific phase transition property to temperature-sensitive hydrogels. For example, poly(*N*-isopropylacrylamide) (PNIPAAm) and poly(*N*,*N*-diethylacrylamide) (PDEAAm) are well-known LCST polymers [19,20]. Methylcellulose (MC), a cellulose polysaccharide derivative and natural polymer, also exhibits LCST properties in aqueous solutions (Figure 2) [21,22]. On the other hand, poly(*N*,*N*-dimethyl(acrylamidopropyl) ammonium propane sulfate), poly(allylurea) (PU), and poly(L-citrulline) copolymer have a typical UCST behavior [23,24]. Additionally, the mixture or copolymerization of poly(acrylamide) (PAAm) and poly(acrylic acid) (PAAc) also have an UCST property (Figure 2) [25].

### 2.2. pH-Sensitive and Electro-Sensitive Hydrogels

pH- and electro-sensitive hydrogels are designed to respond to variations in ionic conditions that frequently accompany pathological states, including the acidic tumor microenvironment.

**Figure 3:**
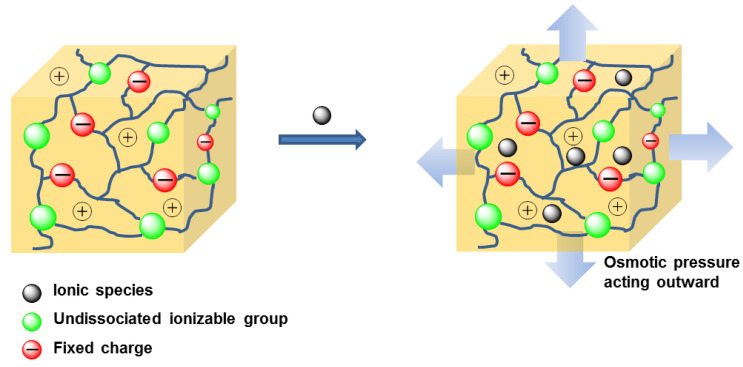
pH-sensitive hydrogel swelling.

Such pH-dependent volume changes are primarily governed by the ionization of polymer chains and electrostatic repulsion, which can be harnessed for selective activation and controlled therapeutic release. Acidic or basic groups of these polymer chains ionize in an aqueous solution of appropriate pH and ionic strength. When the environmental pH rises above the pKa of the polymer chain groups, ionization occurs, resulting in solvent uptake by the hydrogel due to electrostatic repulsions between polymer chains [26]. The process of solvent uptake can also be explained by osmotic pressure. For example, acidic groups bound to polymer chains constituting a hydrogel can release the hydrogen ion (H^+^) in basic solutions. These hydrogen ions subsequently react with OH^−^ ions to form H_2_O. The deprotonated acidic groups of polymers are then compensated by cation influx from outside to maintain charge neutrality. This increase in cation concentration within the hydrogel generates osmotic pressure (i.e., higher osmotic pressure inside the hydrogel than surroundings), leading to hydrogel swelling (Figure 3) [18]. Electro-sensitive hydrogels, composed of polyelectrolytes within ionizable groups distributed throughout their polymer crosslink, exhibit swelling–deswelling properties in response to an applied electric field [2].

**Figure 4:**
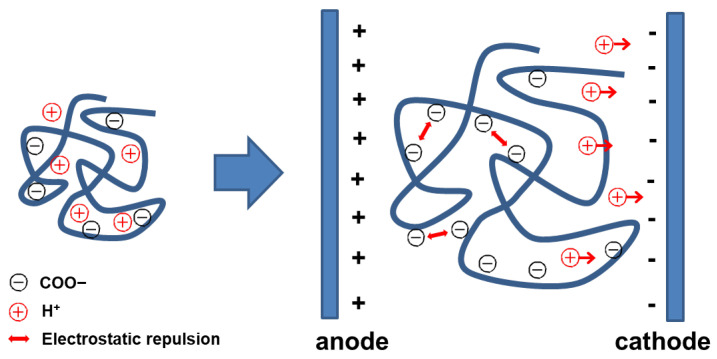
Electro-sensitive hydrogel swelling.

Electro-sensitive hydrogels behave similarly to pH-sensitive hydrogels. For example, in hydrogels containing carboxyl groups placed between anode and cathode electrodes, hydrogen ions inside the hydrogel migrate toward the cathode under applied potential, increasing the –COO^−^ groups inside the hydrogel. These ionized groups induce electrostatic repulsion between polymer chains, causing hydrogel swelling (Figure 4) [27]. Despite their precise external controllability, electro-sensitive hydrogels face practical limitations related to electrode implantation, power supply requirements, and long-term tissue compatibility [28,29]. These constraints currently restrict their clinical translation. Emerging efforts therefore explore wireless stimulation platforms and biointegrated conductive materials to enable minimally invasive electrical modulation in vivo [28].

### 2.3. Biomolecule-Sensitive Hydrogels

**Figure 5:**
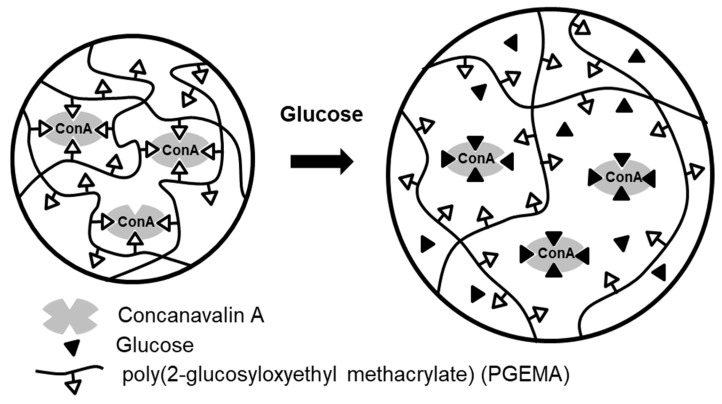
Glucose-sensitive swelling changes in a poly(GEMA)–Con A hydrogel.

**Figure 6:**
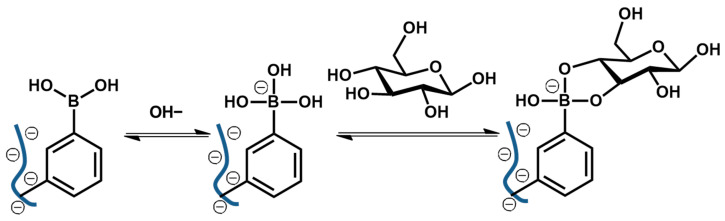
Mechanism of phenylboronic acid–glucose complex formation. The ⊖ symbol indicates the negative charge of the boronate species.

Biomolecule-sensitive hydrogels represent an important class of smart materials capable of responding to specific biological signals through molecular recognition. By incorporating enzymes, lectins, boronic acids, antibodies, or nucleic acid motifs, these hydrogels enable highly selective and biologically relevant responsiveness, extending their utility beyond conventional physicochemical triggers. ConA is a derivative of the carbohydrate-binding protein lectin, modified with four glucose-binding sites. Each binding site of ConA can recognize α-D-mannosyl and α-D-glucosyl residues of carbohydrates [30]. Previously, Nakamae et al. demonstrated that ConA can be used as a crosslinking material in a hydrogel (Figure 5) [31]. In this system, glucose-conjugated hydrogels, i.e., Poly (2-glucosyloxyethyl methacrylate) (PGEMA)-entrapping ConA, exhibited swelling behavior in response to external glucose concentration due to the dissociation of the complex through competitive exchange with environmental glucose. In Gox-conjugated hydrogels, Gox is bound to a poly(amine)-based hydrogel, where environmental glucose is converted to gluconic acid by enzymatic reaction with Gox. This process lowers the microenvironmental pH in the hydrogel due to increased gluconic acid, causing hydrogel to swell as a result of increased ionization of the polymer’s amine groups [32]. As another glucose-sensitive moiety, boronic acid (BA) derivatives exhibit reversible glucose binding and have been extensively investigated [33,34]. In this system, nonionic trigonal BA moieties of anionic polymer become negatively charged tetrahedral boronate esters in the presence of glucose (Figure 6). This charge change causes volumetric expansion of the hydrogel due to the overall negative charge and electrostatic repulsion. Similarly, various bioactive hydrogels modified with specific ligands that recognize biological targets, including nutrients, enzymes, antibodies, or cells, have been developed. Additionally, biomolecule-sensitive hydrogels that adjust their structural composition in response to target biomolecules such as polypeptides, proteins, and nucleic acids have recently attracted considerable attention for their potential biomedical applications.

## 3. Biomedical Applications of Stimuli-Responsive Hydrogels

### 3.1. Drug Delivery Systems

The purpose of drug delivery systems is to control the release rate of therapeutic agents (i.e., kinetics) and to improve the specificity of drug targeting. An effective drug delivery strategy requires transporting pharmaceutical compounds to the appropriate location at an optimal concentration and desired time point [2,35]. In this context, stimuli-responsive hydrogels have emerged as promising drug delivery platforms, as their reversible swelling behavior in response to external stimuli enables spatiotemporally controlled drug release, while their polymeric networks allow efficient encapsulation of biomolecules [36,37].

The swelling ratio and drug loading/release efficiencies of hydrogels are determined by the following equations [38]:

(1) $$\mathrm{Swelling}\;\mathrm{ratio}=\frac{Ws-Wd}{Wd}$$

where *W_d_* is the weight of the dried hydrogel and *W_s_* is the weight of the hydrogel after swelling [39].

(2) $$\mathrm{Encapsulation}\;\mathrm{efficiency}\;(\%)=\frac{\left(m1-m2\right)}{m1}\times 100$$

(3) $$\mathrm{Loading}\;\mathrm{efficiency}\;(\%)=\frac{\left(m1-m2\right)}{\left(m1-m2+m3\right)}\times 100$$

where *m*_1_ is the initial amount of drug loaded in the hydrogel [39,40], *m*_2_ is the amount of free drug, and *m*_3_ is the amount of hydrogel.

(4) Cumulative release (%)=∑Mt/M0×100

where ∑Mt is the total weight of the released drug at time t [41] and *M*0 is the total absorbed in the hydrogel.

**Figure 7:**
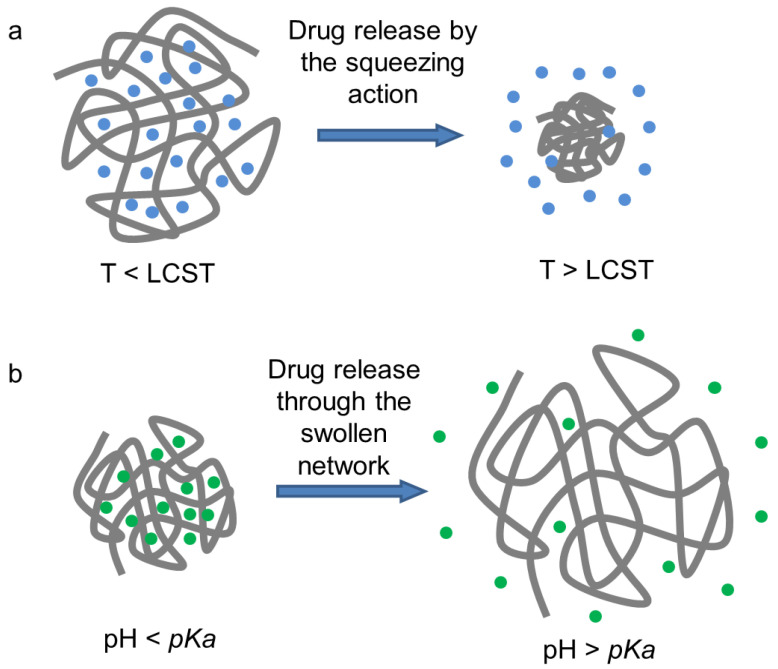
Schematic illustration of drug release from temperature-sensitive and pH-sensitive hydrogels. (**a**) drug release from a temperature-sensitive hydrogel. Below the LCST (T < LCST), the polymer network is swollen and retains drug molecules, whereas above the LCST (T > LCST), the hydrogel collapses and releases the drug through a squeezing action; (**b**) drug release from a pH-sensitive hydrogel. At pH < pKa, the polymer network remains collapsed and retains drug molecules, whereas at pH > pKa, ionization of functional groups causes swelling of the hydrogel network, leading to drug release.

Numerous strategies have been developed to enhance stimuli-responsive hydrogels for controlled drug delivery systems. In thermosensitive hydrogels, drug release primarily occurs through a squeezing action of the hydrogel at a specific temperature (Figure 7a). In the case of LCST hydrogels, as the temperature rises above the LCST, the hydrogel becomes hydrophobic and insoluble, causing it to shrink and release the encapsulated drug. At the molecular level, the squeezing action arises from temperature-induced dehydration of polymer chains above the LCST, leading to disruption of polymer–water hydrogen bonding, enhanced hydrophobic association, and consequent network contraction. This volumetric shrinkage generates internal pressure that expels the encapsulated drug from the hydrogel matrix. An example is the chitosan/glycerophosphate (CS/GP) hydrogel reported by Chenite et al. [42], where chitosan is a pH-dependent cationic polymer that gains thermal sensitivity with the addition of glycerophosphate. The gelation mechanisms of the CS/GP system have been described in previous studies [43,44,45]. Table 1 shows the recent studies on CS/GP temperature-sensitive hydrogels for drug delivery applications. To enhance the thermosensitive properties, various gelling agents have been tested as alternatives to GP [46]. Another widely studied thermosensitive hydrogel for drug delivery is based on poly(*N*-isopropylacrylamide) (PNIPAAm), an LCST polymer with a transition temperature of 32 °C [47]. Recent applications of PNIPAAm-based hydrogels in drug delivery systems are listed in Table 2. Additionally, Yu et al. developed a thermosensitive hydrogel containing poly (ethylene glycol)-modified gold nanoparticles or nanorods crosslinked by α-cyclodextrin (α-CD). The authors evaluated this hydrogel using doxorubicin as a model drug in in vitro release, cytotoxicity, and intracellular uptake studies [39].

For pH-sensitive hydrogels in drug delivery systems, drug release is triggered at a pH above the polymer’s pKa (Figure 7b). For example, carboxylated polymeric hydrogel deprotonates at a high pH, causing anionic carboxyl groups to create electrostatic repulsion within the gel, which swells and releases encapsulated drugs. Piao et al. developed a graphene oxide (GO)–gelatin hydrogel with ~98 wt% water content and a pH sensitivity based on the protonation/deprotonation of gelatine amine groups, achieving a 96% cumulative drug release with fluorescein sodium as a model drug [63]. Heleg-Shabtai et al. designed an anticancer chemotherapeutic drug, gossypol, incorporated into a boronic acid-modified polyacrylamide hydrogel for controlled drug release, leveraging the acidic environment in tumors for targeted release [64,65]. Dual-stimuli hydrogels sensitive to both pH and temperature have also been reported. Ma et al. synthesized a P (*N*,*N*-diethylacrylamide-co-methacrylic acid) (P(DEA-co-MAA)) microsphere using bovine serum albumin (BSA) as a model drug. The system, with a temperature-sensitive PDEA core and a pH-sensitive PMMA shell, achieved drug encapsulation efficiencies from 40% to 70% and varied release rates based on pH conditions [66,67,68]. Guo et al. synthesized a poly (methacrylic acid) (PMAA)-grafted-keratin with pH- and temperature-sensitive properties, showing controlled drug release of rhodamine B (small molecule) and bovine serum albumin (BSA, macromolecule) with high cumulated rates [41]. Shi et al. showed a dual pH- and electro-sensitive system using a bacterial cellulose nanofiber and sodium alginate (SA) hybrid hydrogel. Ibuprofen, known as a nonsteroidal anti-inflammatory drug, was loaded into the hydrogel to evaluate the drug release characteristics depending on the pH and electric field. According to their results, hydrogel swelling ratios ranged from less than 8 times (at pH 1.5) to 13 times (at pH 11.8) and from 8 times (at 0 V) to 14 times (at 0.5 V), and their drug release% for 2 h ranged from less than 10% (at pH 1.5) to 31% (at pH 11.8) and from 10% (at 0 V) to 38% (at 0.5 V), respectively [27]. As a recently reported electro-sensitive hydrogel system for drug delivery, Ying et al. developed a brain-targeting hydrogel by copolymerization of 2-dimethylamino ethylmethacrylate (DMAEMA), sodium 4-vinylbenzene sulfonate (NaSS), styrene (ST), and acrylate-poly (ethylene glycol)-*N*-hydroxysuccinimidylester (ACLT-PEG-NHS). For facilitated penetration of the blood–brain barrier (BBB), they further conjugated angiopep-2, a brain-targeting peptide, to the hydrogel and loaded phenytoin sodium (PHT), an antiepileptic drug, into the hydrogel with ~80% encapsulation efficiency. This system showed efficient antiepileptic effect in the amygdala kindling model [28]. While thermosensitive systems offer minimally invasive administration, their burst release and inconsistent in vivo degradation remain concerns. In contrast, pH-sensitive systems provide microenvironment selectivity but may suffer from limited pH gradients under physiological conditions. Comparative studies evaluating long-term toxicity and pharmacokinetics across these platforms remain scarce and warrant systematic investigation [1,2,10]. Table 3 summarizes the main advantages, key limitations and safety concerns, and representative examples of thermo-, pH-, electro-, and biomolecule-responsive hydrogels used in drug delivery applications.

### 3.2. Gene Delivery System

In gene therapy, stimuli-responsive hydrogels offer a non-viral strategy for localized and sustained nucleic acid delivery while minimizing cytotoxicity and immune activation. Recent designs increasingly emphasize microenvironment-adaptive release and cell-responsive degradation, enhancing transfection efficiency in complex 3D tissues. Despite these advances, efficient intracellular delivery of nucleic acids remains a major challenge in gene therapy. However, DNA is a negatively charged, hydrophilic molecule, which makes it difficult to penetrate the cell membrane due to the membrane’s negative charge and hydrophobic nature. For this reason, various polymeric hydrogels have been developed as non-viral carriers for gene delivery, providing an alternative to virus-based materials, which can trigger immune responses.

For effective gene delivery, polymeric hydrogels should be designed with consideration for non-cytotoxicity, efficient transfection property, and biodegradability. Positively charged polymers such as polyethyleneimine (PEI), Poly L–lysine (PLL), and chitosan have been used for gene delivery due to their high transfection efficiency, though they still pose cytotoxicity challenges [69]. To improve biocompatibility, polyethylene glycol-conjugated PEI or degradable PEI have been explored, though these modified forms generally have reduced therapeutic efficacy compared to standard PEI [13,54]. Kim et al. developed a polyplex hydrogel, low-molecular-weight PEI (~800 Da) and poly(organophosphazene) conjugates, achieving high cell viability. After loading siRNAs targeting VEGF (vascular endothelial growth factor) and cyclin B1 (a key mitotic regulator protein), they demonstrated approximately 60% of gene silencing effect for VEGF and a sustained anti-tumoral effect for up to 4 weeks with cyclin B1 [70]. Recently, Krebs et al. showed alginate and collagen-based hydrogels for siRNA delivery. Alginate, a negatively charged polysaccharide biopolymer, enables retention and controlled release of siRNA from the gel. To further control siRNA release, positively charged polymers were added, and the gene silencing effects of GFP were evaluated in HEK293 cells [71]. More recently, Yang et al. developed a PNIPAAm/LDH (layered double hydroxides) hybrid hydrogel as a siRNA delivery carrier for targeting several negative regulators of tissue homeostasis in cartilaginous tissues, achieving an 82–98% gene silencing effect [72]. While cationic polymers such as PEI offer high transfection efficiency, their cytotoxicity and nonspecific interactions with serum proteins remain major limitations for clinical translation. Surface modification strategies, including PEGylation or charge shielding, can improve biocompatibility but often compromise gene delivery efficiency [10,69,70]. In addition, long-term in vivo safety, immune activation, and reproducibility across different tissue environments remain insufficiently characterized. Systematic comparative studies evaluating transfection performance, degradation behavior, and long-term toxicity are therefore essential to facilitate the clinical advancement of hydrogel-based gene delivery platforms [10].

### 3.3. Biosensors

Beyond therapeutic delivery, stimuli-responsive hydrogels have emerged as key components in biosensing platforms, where their volume, optical, or electrical changes can be transduced into measurable signals. Advances in hydrogel microfabrication and molecular recognition have expanded their applications in multiplexed and real-time biosensing. In biosensing applications, the integration of hydrogel design with robust detection strategies is essential for effective signal transduction. This review focuses on biological targeting strategies and recently developed stimuli-responsive hydrogels, excluding detailed descriptions of fabrication processes or transducer detection methods. Among various biosensors, glucose sensors have been extensively studied over the past decades. Early research focused on using the enzyme glucose oxidate (Gox) to sense glucose, using electrical and optical transduction by enzyme reactions.

The following reaction illustrates the Gox process for glucose sensing.

Glucose + Gox/FAD → Gluconolactone + Gox/FADH_2_

Gox/FADH_2_ + O_2_ → Gox/FAD + H_2_O_2_

H_2_O_2_ → O_2_ + 2H^+^ + 2e^−^

As shown, the electron produced by the Gox–glucose reaction is used as an electrical or optical signaling mediator. Kotanen et al. developed an electroconductive poly (hydroxyethyl methacrylate)-polypyrrole hydrogel thin film coated onto the electrodes, with Gox immobilized within the hydrogel. This system generated electric signals by measuring the rate of hydrogen peroxide produced by Gox enzyme activity in the presence of glucose, achieving a sensitivity from 11.6 to 21.2 nA/mM at a charge density of 1.0 mC/cm^2^ and a response time of 50 s in the linear range of 1 to 15 mM glucose [29]. In another example, Zhai et al. used a platinum nanoparticle–polyaniline hydrogel as a highly sensitive glucose sensor. In this system, platinum nanoparticles acted as catalysts for the electro-oxidation of the produced H_2_O_2_. This sensor achieved 96.1 µA/mM·cm^2^ sensitivity with a fast response time (3 s) and a linear range of 0.01 to 7 mM with a low detection limit of 0.7 µM [73]. However, enzyme-based glucose sensors still face challenges, as enzymes are sensitive to environmental factors, are prone to denaturation, require oxygen and a redox mediator, consume glucose, and produce H_2_O_2_. To overcome these limitations, non-enzymatic glucose sensors have been developed. Phenylboronic acid (PBA) has recieved considerable interest due to its reversible binding property with glucose. For example, Zhou et al. used poly-PBA as a non-enzymatic glucose sensing material, integrating it to graphene and PNIPAM to form a microgel crosslinked with *N*,*N*′-methylene bis(acrylamide). This microgel exhibited glucose-dependent swelling–deswelling that altered the property of the microgel due to changes in the functional groups on the graphene sheets [74]. To enhance the sensitive binding of PBA to glucose, Zhang et al. recently added a volume-resetting reagent, 1,3-diol, to the hydrogel, eliminating the coexistence of 2:1 and 1:1 PBA-glucose complexes, resulting in a linear glucose-response property and fast kinetics (~7 min for 90% reaction with glucose) within the clinical glucose range [75].

In addition to glucose sensing, hydrogels sensitive to nucleic acid or protein/antibody have also been widely studied. For nucleic acid detection, various target oligonucleotides were directly detected by fluorescence signal from changes in shapes, structural color or graphical codes through hybridization assays using specific oligonucleotide probe-functionalized poly (ethylene glycol) diacrylate (PEGDA) hydrogels [76,77,78,79,80,81,82]. PEGDA hydrogels have also been used in protein detection assays. Appleyard et al. developed a high-throughput multiplexed protein quantification assay for detecting three cytokines, including interleukin-2 (IL-2), interleukin-4 (IL-4), and tumor necrosis factor alpha (TNFα), using monoclonal antibody-conjugated PEGDA hydrogel [83]. Additionally, Ye et al. and Srinivas et al. used aptamers, short nucleic acid sequences, conjugated onto PEGDA hydrogel, demonstrating sensitive detection of target proteins such as thrombin, adenosine, and immunoglobulin E antibodies [84,85]. Building on these concepts, molecularly responsive aptamer-functionalized hydrogels have been applied for continuous plasmonic biomonitoring of therapeutic proteins and immune checkpoints, while photonic PEGDA hydrogels combining structural and fluorescent color have been reported as dual-mode encoded biosensing platforms [86,87,88].

### 3.4. Tissue Engineering

In tissue engineering, hydrogels are no longer viewed solely as passive scaffolds but as dynamic matrices capable of instructing cell behavior. Stimuli-responsive hydrogels enable temporal control over mechanical properties, degradation, and biochemical signaling, thereby supporting tissue maturation and regeneration. Recent advances have demonstrated that stimuli-responsive nanogel coatings integrated with grafted polymer brush architectures can dynamically regulate cell adhesion and morphology in response to external triggers. For example, thermo-responsive brush systems incorporating functional nanoparticles have enabled controlled cell sheet harvesting through temperature-mediated modulation at the material interface [89,90]. These developments highlight a transition from conventional bulk scaffold design toward interface-responsive biomaterials capable of fine-tuning cell–material interactions. To design hydrogel scaffolds for tissue engineering, a variety of technologies such as electrospinning, lyophilization, gas foaming, stereolithography, micro-molding, porogen leaching, and laser sintering have been developed to fabricate 3D microporous structures [91,92,93,94,95,96,97,98]. More recently, many studies have focused on fabricating hydrogels with microchannel-like pore structures because human tissues consist of microchannel networks such as microvascular networks and nerve bundles, which are critical for the transport of nutrients, oxygen, metabolic waste, and neuronal signals [16,17,99,100].

Among the various hydrogel systems, gelatin methacrylate (Gel-MA) is particularly attractive for tissue engineering applications. Gelatin contains natural cell-binding motifs, Arg-Gly-Asp (RGD) and matrix metalloproteinase (MMP)-sensitive degradation site, and it is inexpensive while supporting long-term cell viability. Additionally, the methacrylate group in Gel-MA allows it to be photo-crosslinked to form a porous hydrogel [101,102,103,104,105]. Recently, Hammer et al. developed a calcium alginate (Ca-Alg)-based microfiber-encapsulated Gel-MA system to fabricate 3D hydrogels with microchannel-like pore structures. Microchannels were generated by dissolving Ca-Alg fibers using ethylenediaminetetraacetic acid (EDTA), a calcium chelator, resulting in the formation of cylindrical lumen structures within the scaffold [106]. In another study, Chen et al. developed a hydrogel scaffold designed to engineer organized, multi-layered muscle tissue for implantation, aiming to repair or restore the function of damaged tissues [107,108,109].

## 4. Conclusions and Perspectives

Stimuli-responsive hydrogels, also known as smart hydrogels, possess significant potential for diverse biological and biomedical applications due to their reversible swelling–deswelling behavior and volume phase transitions in response to physicochemical and biological cues such as pH, ionic strength, temperature, and molecular recognition. Their tissue-like physical properties, high water content, and capacity for controlled encapsulation and release of bioactive molecules underpin applications in drug and gene delivery, biosensing, and tissue engineering.

In recent years, research in this field has progressed beyond conventional swelling–deswelling-based systems toward application-driven and spatiotemporally programmable hydrogel platforms, enabling localized and on-demand regulation of biological microenvironments. Advances in molecular design, fabrication strategies, and material engineering have led to injectable, in situ-forming, and multi-stimuli-responsive hydrogels with enhanced functional precision. Despite these advances, several challenges remain that continue to limit clinical translation, including insufficient control over hydrogel dimensions, swelling–deswelling kinetics, and long-term structural stability. Diffusion-limited response in bulk hydrogels, inconsistent in vivo degradation leading to burst release or incomplete payload delivery—as observed in systems such as CS/GP and PNIPAAm—and limited scalability of microfabrication for GMP production represent key technical barriers. Addressing these limitations will depend on continued advances in structural and molecular engineering. 

Enhancing response kinetics in PNIPAAm/PDEAAm-based systems through hierarchical porosity engineering or nanoscale architectures may help decouple volumetric swelling from mechanical stiffness. Likewise, improving multi-stimuli orthogonality through advanced copolymer design, including PNIPAAm/PAAc hybrids with tunable LCST/UCST behavior, may reduce crosstalk under physiologically relevant conditions. Strengthening translational feasibility will further require standardized fabrication approaches, such as Gel-MA/Ca-Alg microchannel architectures, together with long-term pharmacokinetic evaluation of siRNA/polyplex hydrogels in large-animal models to establish clinically meaningful release profiles and safety.

Overall, continued interdisciplinary research integrating materials science, biology, and translational medicine—guided by quantitative models of hydrogel–tissue interactions—will be essential to advance stimuli-responsive hydrogels toward clinically viable platforms for precision biomedical applications.

## Figures and Tables

**Table 1:** CS/GP thermosensitive hydrogel for drug delivery applications.

CS			Drug	Objective of Study	Ref.
Mw (kDa)	Deacetylation (%)	Formulation (CS/GP)			
Medium mw	n.a.	1.8%/7.27% (*w*/*w*)	Chlorpheniramine maleate	In vitro controlled release	[48]
1360	75.4	2.0%/5% (*w*/*v*)	Adriamycin or 6-mercaptopurine	In vitro release of hydrophilic and hydrophobic drugs	[49]
n.a.	95	1.4–2.2%/8–16% (*w*/*w*)	Venlafaxine hydrochloride	Release mechanism investigation	[50]
228.7	95	1.8%/3.6% (*w*/*w*)	Paclitaxel	Intratumoral administration	[51]
300	85	1.53% *w*/*w*	Camptothecin	Sustained intratumoral release	[52]
<310	<75	1.5%/0.97–14.2% (*w*/*v*)	Ellagic acid	Anti-tumor evaluation (brain cancer cells)	[53]
161	80	2.0%/0.01% (*w*/*v*)	Doxorubicin	Combination with vaccinia virus vaccine	[54]
200	86	1.67%/9.33% (*w*/*v*)	Doxorubicin	In vivo antitumor evaluation	[55]
n.a.	90	1.8%/5.7% (*w*/*v*)	Cytarabine (±liposomes)	Sustained in vivo delivery (free or loaded)	[56]
200–700	74 or 99	1%/23% (*w*/*w*)	Meglumine antimoniate (±microspheres)	Sustained in vivo delivery	[57]

n.a.: not available.

**Table 2:** PNIPAAm-based thermosensitive hydrogels for drug delivery applications.

Hydrogel	Component	Drug	Loading Capacity (%)	Objective of Study	Ref.
PNIPAAm–PAMAM dendrimer hydrogel	PNIPAAm (300 mg); PAMAM (0, 30, 90 mg)	Paracetamol	39–62	Drug-loading and release evaluation	[58]
PNIPAAm-co-PLLA-b-PEG-b-PLLA	PNIPAAm; PLLA; PEG (Mn 3350 Da)	Avastin; Lucentis (anti-VEGF agents)	75–85	Anti-tumor activity in HUVEC cells	[59]
PNIPAAm-g-PAAc	NIPAAm (Mw 44.25 kDa); PAAc (2.33–4.92 mmol)	Epinephrine	–	In vivo ophthalmic delivery (glaucoma therapy)	[60]
PNIPAAm-b-PPS-b-PDMA	PNIPAAm; PPS (150 molar ratio); PDMA (60 molar ratio)	Nile red (model hydrophobic drug)	–	ROS-responsive drug release (in vitro/in vivo)	[61]
(PNIPAAm-PCL)_2_-PEG	PNIPAAm; PCL (Mw 4398); PEG (Mw 400–2000)	Naltrexone hydrochloride	–	Controlled drug release (in vitro/in vivo)	[62]

**Table 3:** Advantages and limitations of stimuli-responsive hydrogels for drug delivery.

Stimulus Type	Main Advantages	Key Limitations/Safety Concerns	Representative Examples
Thermosensitive (LCST)	In situ gelation after injection, minimally invasive local depot	Burst release, diffusion-limited deswelling, variable in vivo degradation and retention	CS/GP local anticancer gels; PNIPAAm-based depots
pH-sensitive	Tumor/inflammation microenvironment selectivity, suitable for oral/topical use	Limited physiological pH gradients, potential mucosal irritation and scarce long-term PK/toxicity data	GO–gelatin hydrogels; PBA–PAAm gossypol systems
Electro- sensitive	Precise, switchable on–off control of release	Requires electrodes and power supply; possible tissue damage and chronic inflammation; limited long-term data	Angiopep-conjugated electro- responsive hydrogel (antiepileptic)
Biomolecule- sensitive	High specificity via enzymes, sugars, antibodies, aptamers	Complex design; variable target expression; potential immune responses to bio-components	GEMA–ConA glucose-responsive gels; PBA-based glucose sensors

## Data Availability

No data was used for the research described in the article.

## Abbreviation

The following abbreviations are used in this manuscript:

Abbreviation	Full Form
CS/GP	chitosan/glycerophosphate
LCST	Lower Critical Solution Temperature
UCST	Upper Critical Solution Temperature
PNIPAAm	poly(*N*-isopropylacrylamide)
PAMAM	poly(amidoamine) dendrimer
PLLA	poly(L-lactic acid)
PEG	poly(ethylene glycol)
VEGF	Vascular Endothelial Growth Factor
PAAc	poly(acrylic acid)
PPS	poly(propylenesulfide)
PDMA	poly(*N*,*N*-dimethylacrylamide)
ROS	Reactive oxygen species
PCL	poly(ε-caprolactone)
